# MR-guided Focused Ultrasound Facilitates Sonodynamic Therapy with 5-Aminolevulinic Acid in a Rat Glioma Model

**DOI:** 10.1038/s41598-019-46832-2

**Published:** 2019-07-18

**Authors:** Sheng-Kai Wu, Marc A. Santos, Stuart L. Marcus, Kullervo Hynynen

**Affiliations:** 1 Physical Sciences Platform, Sunnybrook Research Institute, Toronto, ON Canada; 2 Department of Medical Biophysics, University of Toronto, Toronto, ON Canada; 3 Sun Pharmaceutical Industries Inc., Princeton, New Jersey United States; 4 Institute of Biomaterials and Biomedical Engineering, University of Toronto, Toronto, ON Canada

**Keywords:** CNS cancer, Biomedical engineering

## Abstract

Glioblastoma multiforme (GBM) continues to have a dismal prognosis and significant efforts are being made to develop more effective treatment methods. Sonodynamic therapy (SDT) is an emerging modality for cancer treatment which combines ultrasound with sonosensitizers to produce a localized cytotoxic effect. It has long been known that ultrasound exposure can cause both thermal and non-thermal bioeffects and it remains an open question to what degree does temperature impact the efficacy of SDT. In order to optimize the ultrasound parameters of SDT, transcranial MRI-guided focused ultrasound (MRgFUS) and real-time MRI thermometry were used to monitor the therapy in a rat brain tumor model. Experiments were performed using a C6 intracranial glioma tumor model in 37 male Sprague Dawley rats. Treatments were performed about 7 days following tumor implantation when the tumor reached 1–3 mm in diameter as determined by MRI. 5-aminolevulinic acid (5-ALA) was injected at a dose of 60 mg/kg six hours before sonication. MRgFUS at 1.06 MHz was delivered continuously at an *in situ* spatial-peak temporal-average intensity of 5.5 W/cm^2^ for 20 min. MR thermometry was acquired to monitor the temperature change in the brain during sonication. The tumor growth response for animals receiving 5-ALA alone, FUS alone, 5-ALA + FUS and a sham control group were evaluated with MRI every week following treatment. During 20 min of MRgFUS at 5.5 W/cm^2^, the temperature within the targeted brain tumor was elevated from 32.3 ± 0.5 °C and 37.2 ± 0.7 °C to 33.2 ± 0.9 °C and 38.4 ± 1.1 °C, respectively. Both the tumor growth inhibition and survival were significantly improved in the 5-ALA + FUS group with 32 °C or 37 °C as the starting core body (rectal) temperature. 5-ALA alone and FUS alone did not improve survival. These promising results indicate that relatively low power continuous wave transcranial MRgFUS in conjunction with 5-ALA can produce an inhibitory effect on rat brain tumor growth in the absence of thermal dose. Further investigation of the ultrasound parameters is needed to improve the therapeutic efficacy of MRgFUS and 5-ALA.

## Introduction

Glioblastoma multiforme (GBM) is the most common and malignant brain tumor^1^. The optimal therapeutic regimen for glioblastoma is not yet defined. Surgical intervention remains an essential component of brain tumor treatment when it is possible; however, reducing the degree of intervention has always been recognized as beneficial to patients in terms of outcome and quality of life. External radiotherapy, such as stereotactic radiosurgery (SRS), is one of the promising methods to precisely target and destroy the tumor non-invasively, but the risk and adverse effects are still inevitable^2^. In addition, several chemotherapeutic agents were developed to treat GBM, such as temozolomide (TMZ), bevacizumab, cisplatin and so on. TMZ is the only chemotherapeutic agent which has convincingly proven to prolong survival by 2 months for GBM patients^3^. Despite vigorous surgery following adjuvant chemotherapy and radiotherapy, the median survival is only 15 months after diagnosis and patients with GBM have a 5-year survival rate less than 5%. Hence, the development of new strategies for defeating brain cancer is paramount.

Photodynamic therapy (PDT) is one promising approach to treat tumors under certain conditions. Namely, by using an appropriate wavelength of laser light and a photosensitizer as an energy absorber, localized cell death can be achieved. However, PDT is not effective for the treatment of deep-seated tumors due to the poor penetration depth of light^4^. Three decades ago, several hematoporphyrin derivatives were found to induce significant cell damage under ultrasound exposure *in vitro*^5,6^. More recently it was shown that some photosensitizers can be also used as sonosensitizers to absorb energy from ultrasound in order to potentiate tumor cell death in animal models, a so-called sonodynamic therapy (SDT)^7,8^. The significant advantage of SDT over PDT is that ultrasound can be well-focused and penetrate deep into the target tissue^9^. Therefore, SDT overcomes the major limitation of PDT while maintaining its non-invasiveness.

SDT has yielded promising anticancer effects both *in vitro* and *in vivo*^10^. One possible mechanism of SDT is the generation of reactive oxygen species (ROS) through the simultaneous combination of ultrasound and a sonosensitizer^11^. Aminolevulinic acid hydrochloride (5-aminolevulinic acid HCl; 5-ALA) is one such sonosensitizer and is a precursor of fluorescent protoporphyrin (PPIX). The exogenous administration of 5-ALA leads to a high degree accumulation of PPIX in epithelial tissues and tumor cells. Due to its fluorescent properties, 5-ALA has been used for tumor resection guidance. 5-ALA has also been used in PDT for human glioma treatment^12^. Nevertheless, the main challenge for PDT in treating deep-seated tumors is the placement of an indwelling optical fiber directly adjacent to the target tissue. In contrast, focused ultrasound (FUS) can deliver and concentrate ultrasound energy into a small and deep-seated brain region even through an intact human skull^13^. Upon ultrasound irradiation, the temperature change of the sonicated tissues can be non-invasively monitored by magnetic resonance imaging (MRI) thermometry^14^. Some studies have suggested that the thermal effect is not a key factor for determining the efficacy of SDT^15^; however, others have suggested that tumor temperature might affect the efficacy SDT if the tissue enters the hyperthermia regime due to the enhanced cytotoxic effect of porphyrin derivatives and hyperthermia^16^.

In this work, MRI-guided FUS (MRgFUS) was combined with a sonosensitizing therapeutic agent (5-aminolevulinic acid, 5-ALA) to perform SDT in an intracranial rat glioma model. MR thermometry was used to monitor the temperature change during the treatment at two resting core body temperatures, namely 32 °C and 37 °C. These were chosen to investigate the effect of absolute temperature elevation as measured during the SDT sonication on the resulting tumor control capability and to compare the results with previous literature^15^. Single point and multi-point exposures were also examined in an effort to elucidate the impact of treatment parameters for SDT.

## Results

### Sonodynamic therapy in normal brain tissue at different ultrasound intensities

**Figure 1:**
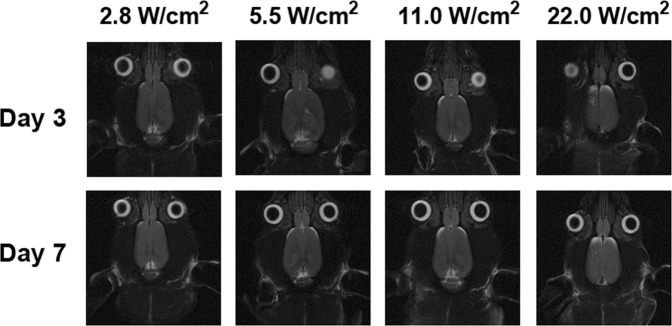
Sonodynamic therapy was applied to normal brain tissues with different intensities for 20 min to determine the suitable intensity for sonication. A thermal lesion was observed on day 3 and day 7 only with the highest intensity applied, 22 W/cm^2^. The intensities from 2.8 to 11 W/cm^2^ (I_SPTA_) did not cause any lesion in the brain.

We first examined the power level of continuous wave MRgFUS for the SDT sonication. Four different I_SPTA_ levels were investigated during a 20 min sonication and the animals were followed up by MRI on day 3 and day 7 following the treatment. Thermal damage near the surface of the brain was only observed following MRgFUS at the highest I_SPTA_ level, 22 W/cm^2^, as seen in Figure 1. For the other three intensity levels, no obvious damage was observed in the normal brain either on day 3 or day 7. An I_SPTA_ of 5.5 W/cm^2^ was selected for the treatment efficacy experiments because under this exposure condition, the brain temperature was elevated by approximately 2 °C which was found to be suitable for the experimental design of this study.

### Real-time temperature responses during FUS exposure

**Figure 2:**
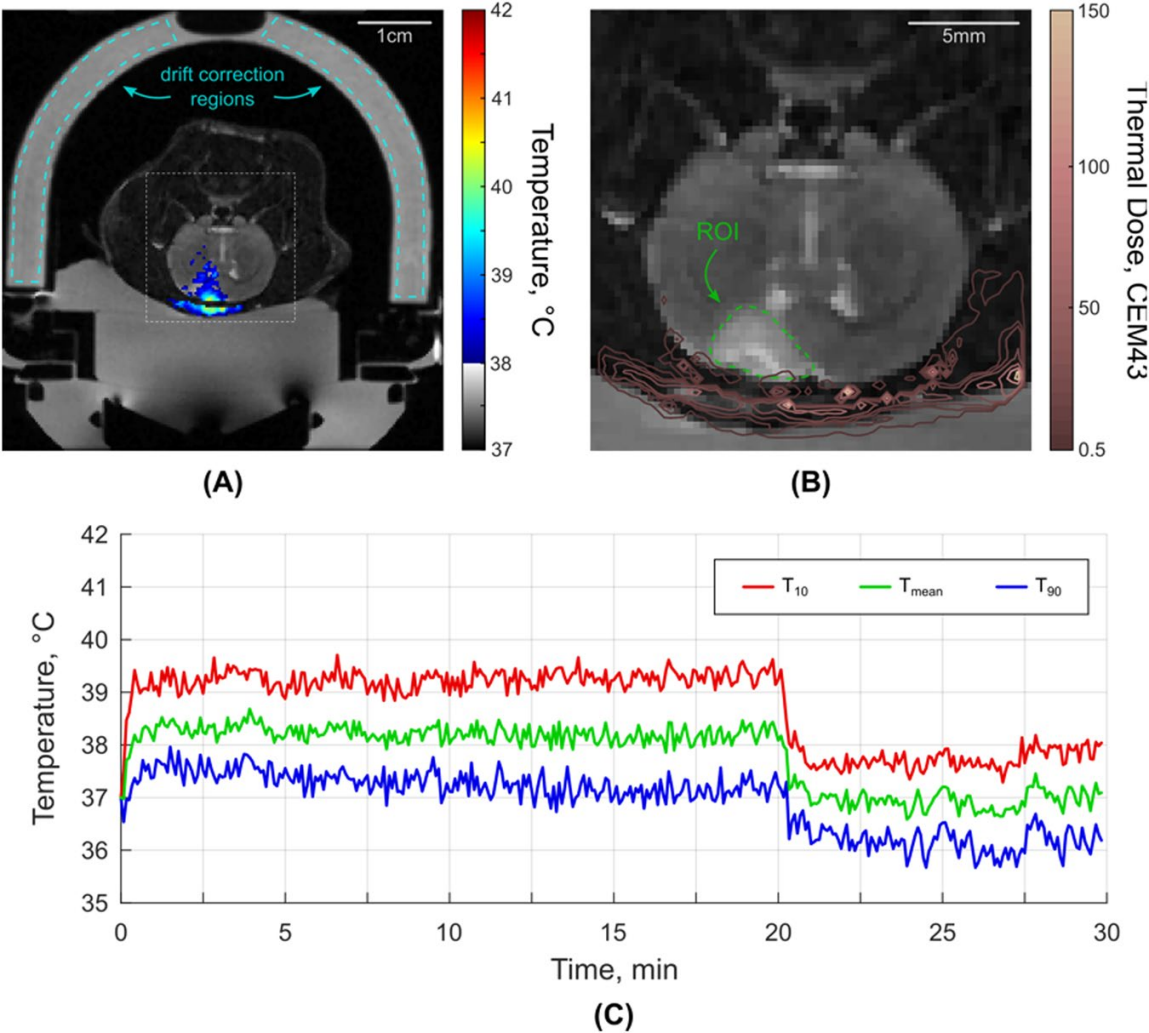
(**A**) MR thermometry overlaid in color upon a T2-weighted anatomical image during MRgFUS + SDT at an I_SPTA_ of 5.5 W/cm^2^. The image regions used for drift correction are outlined in blue. (**B**) Square inset from (**A**) showing thermal dose contours following the sonication in which it is clear that the tumor and healthy brain tissue were not exposed to significant levels of thermal dose confirming that these exposure conditions were not thermally significant. The region-of-interest for monitoring the tumor temperature is outlined in green. (**C**) The temperature profile in the tumor during MRgFUS + SDT from 37 °C core body temperature. The mean temperature within the tumor ROI is shown in green, as well as the temperatures that 90% (blue) and 10% (red) of the region exceeds.

**Figure 3:**
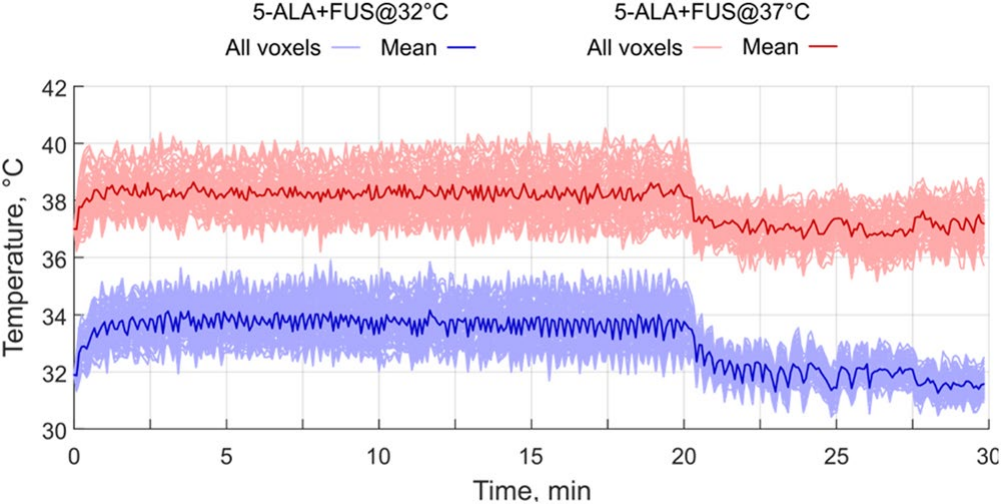
MR thermometry curves during 20 min of MRgFUS at an I_SPTA_ of 5.50 W/cm^2^ for SDT at different starting core (rectal) body temperatures. The temperature profile in the tumor during MRgFUS SDT with a starting body temperature of 32 °C is shown in blue and 37 °C is shown in red.

Figure 2 illustrates the temperature profile in the tumor area from a starting core body temperature of 37 °C. The accumulated thermal dose in the tumor did not exceed 0.5 CEM43 in any of the sonications which suggests that the ultrasound parameters used in this study did not expose the healthy brain or the tumor to hyperthermia conditions. The tumor temperature during SDT was found to elevate from 32.3 ± 0.5 °C and 37.2 ± 0.7 °C at the start of the sonication to 33.2 ± 0.9 °C and 38.4 ± 1.1 °C, respectively over 20 min at a fixed I_SPTA_ of 5.5 W/cm^2^. Table 1 and Figure 3 summarize the data of heating and the accumulated thermal dose during sonication.

**Table 1:** Summary of heating during MRgFUS SDT.

Group	Animal Weight (g)	Baseline Temperature (°C)	T_mean_	T_10_	T_90_	Max Temperature Rise	Median Thermal Dose
Group 32 °C (n = 5)	395	31.6	33.4 ± 0.3	34.3 ± 0.4	32.5 ± 0.3	3.4	<0.5
	385	32.0	31.9 ± 0.4	32.7 ± 0.4	31.2 ± 0.4	1.5	<0.5
	333	33.0	33.9 ± 0.3	34.5 ± 0.3	33.2 ± 0.3	2.2	<0.5
	343	32.6	32.5 ± 0.5	33.4 ± 0.4	31.7 ± 0.7	1.5	<0.5
	334	32.5	34.1 ± 0.4	35.4 ± 0.5	32.9 ± 0.5	3.7	<0.5
Mean ± SD	358 ± 30	32.3 ± 0.5	33.2 ± 0.9	34.1 ± 1.0	32.3 ± 0.8	2.5 ± 1.0	<0.5
Group 37 °C (n = 5)	324	36.0	36.9 ± 0.2	37.9 ± 0.2	35.9 ± 0.4	2.3	<0.5
	305	37.8	39.6 ± 0.3	40.0 ± 0.4	38.7 ± 0.3	2.9	<0.5
	286	37.4	39.2 ± 0.6	40.8 ± 0.6	38.1 ± 0.7	4.7	<0.5
	293	37.2	37.6 ± 0.5	39.0 ± 0.5	36.8 ± 0.5	2.3	<0.5
	327	37.4	38.9 ± 0.7	40.1 ± 0.9	37.4 ± 0.7	4.4	<0.5
Mean ± SD	307 ± 18	37.2 ± 0.7	38.4 ± 1.1	39.6 ± 1.1	37.4 ± 1.1	3.3 ± 1.2	<0.5

### Sonodynamic therapy inhibits tumor growth and improves survival

**Figure 4:**
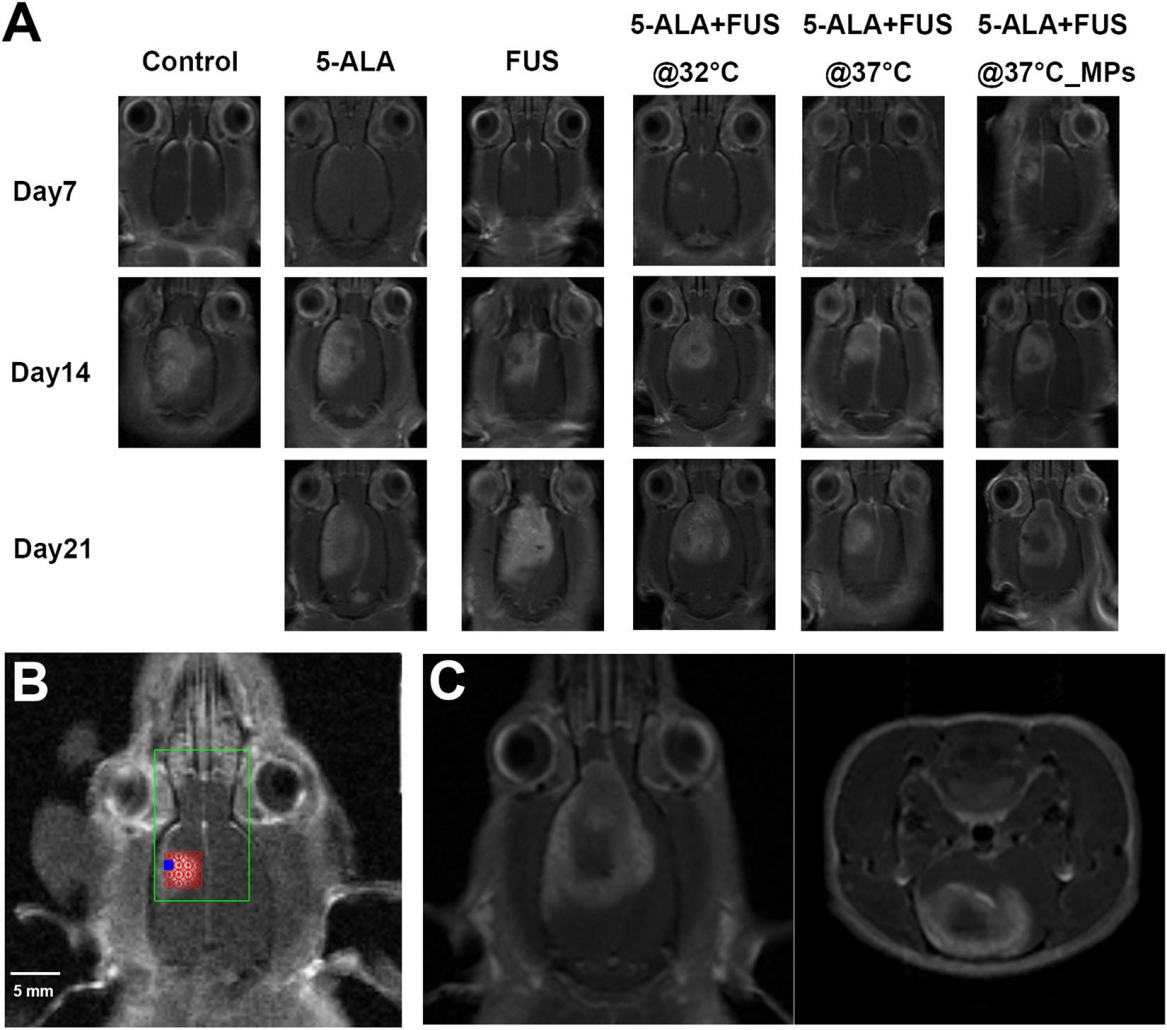
(**A**) The representative T1-weighted images show tumor growth of different groups on day 7, 14, and 21 after the tumor implantation. The treatment was performed on day 7. Note that no control rats survived over Day 21. (**B**) To treat a larger volume of the tumor, a 16-points sonication was exploited to cover the tumor. (**C**) A representative image in the group of 16-points treatment showed the coronal and axial planes of the tumor on Day 28. In the center of the tumor, a non-enhancing lesion was found in accord with the area that had been sonicated.

**Figure 5:**
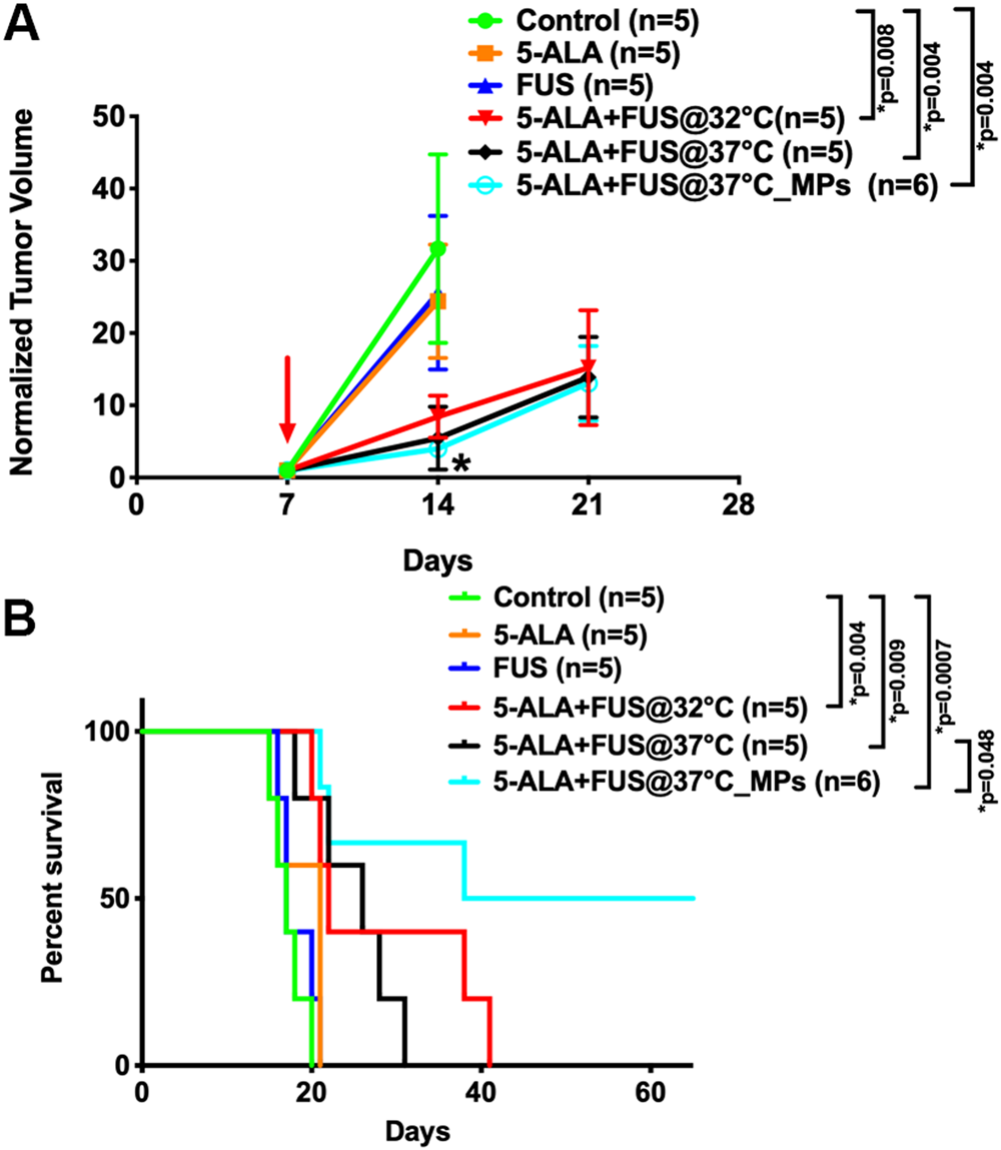
(**A**) Normalized tumor growth curve was shown every week after tumor implanted and the treatment was executed on Day 7. T1-weighted images were used to quantify volume using MIPAV software (NIH). There were six groups: Control (no treatment), 5-ALA, FUS, 5-ALA + FUS@32 °C, 5-ALA + FUS@37 °C, and 5-ALA + FUS@37 °C_MPs. Regardless of which baseline temperature is, sonodynamic therapy markedly inhibited the tumor growth as compared to control, 5-ALA alone, and FUS alone. Data are presented as mean ± SD. *Represents p < 0.05. (**B**) Kaplan-Meier survival curves of tumor-bearing rats with different therapeutics. Sonodynamic therapy significantly increased the lifespan of tumor-bearing rats.

Figure 4A shows the representative coronal images of tumor profile on day 7, 14 and 21. There was no day 21 images for the control group due to the lack of animal survival in this group. On day 14, there are no significant differences in normalized tumor volume among control, 5-ALA and FUS alone groups, whereas the differences between all SDT groups and the previous conditions were found to be statistically significant (Figure 5) (control vs. 5-ALA + FUS@32 °C, p = 0.008; control vs. 5-ALA + FUS@37 °C, p = 0.004; control vs. 5-ALA + FUS@37 °C_MPs, p = 0.004). There is no statistical difference between SDT with 32 °C and 37 °C body temperature baseline (p = 0.65). In the survival data, all SDT groups have a better lifespan improvement than the control group (control vs. 5-ALA + FUS@32 °C, p = 0.004; control vs. 5-ALA + FUS@37 °C, p = 0.009; control vs. 5-ALA + FUS@37 °C_MPs, p = 0.0007); moreover, a significant difference can be found between 5-ALA + FUS@37 °C and 5-ALA + FUS@37 °C_MPs (p = 0.048). Figure 4B demonstrates the treatment plan for the multiple-point sonication. In Figure 4C, the MR image on Day 28 showed a large non-enhanced volume consistent with the sonicated region.

### Histological examination on the antitumor effect induced by SDT

**Figure 6:**
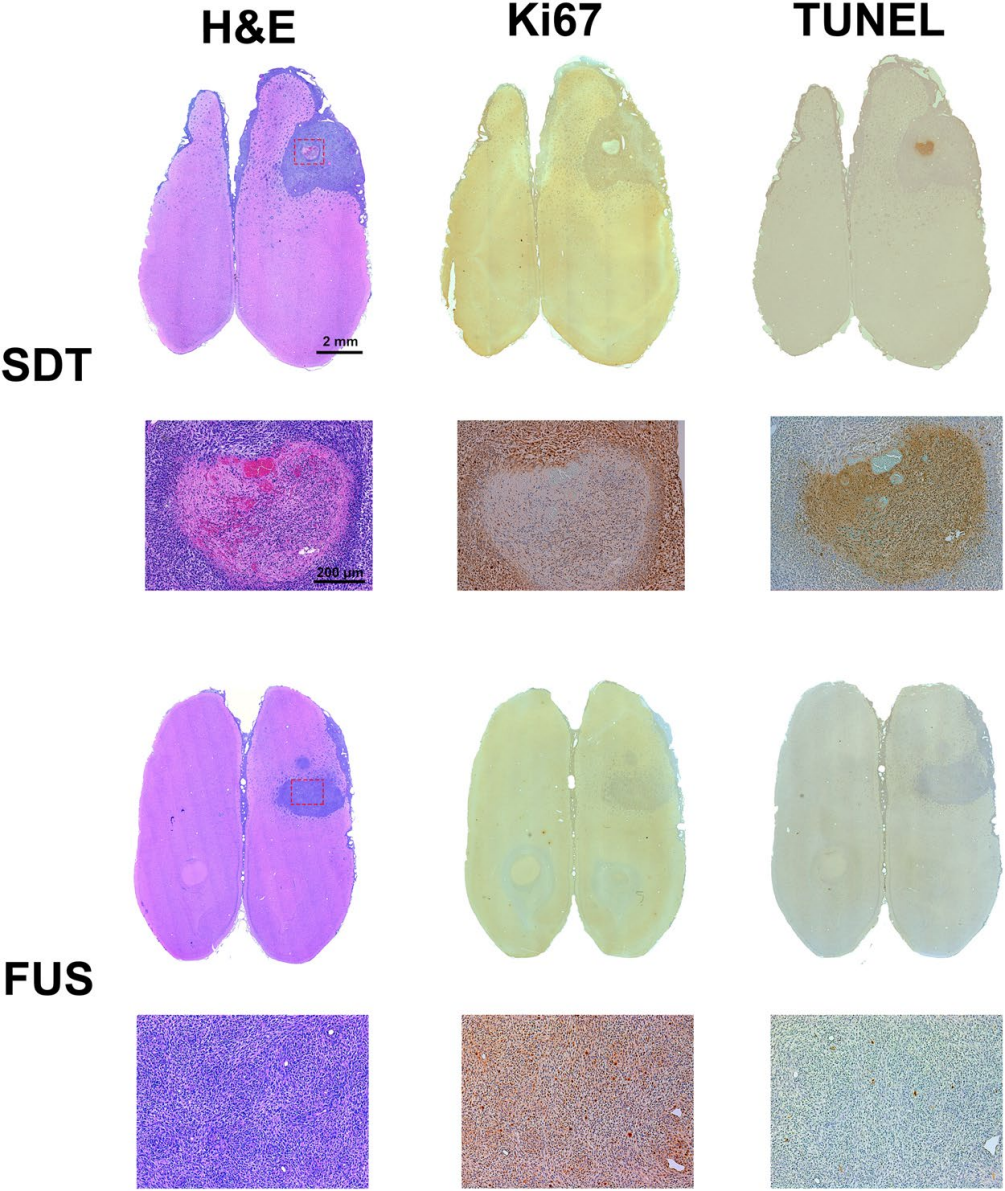
Histology showed the tumor-killing effects of sonodynamic therapy in the rat brain. H&E showed the structures of the brain tumor and the targeted area. Ki67 expression was associated with cell proliferation. Less Ki67 expression was found in the area treated with 5-ALA in conjunction with single-point MRgFUS sonication. TUNEL (Terminal deoxynucleotidyl transferase dUTP-mediated nick-end labeling) staining indicated the apoptotic cells caused by sonodynamic therapy. The less Ki67 expressing area matched the TUNEL overexpressed area. However, the animal received MRgFUS alone demonstrated no damage within the tumor area.

To further determine the mechanisms behind the antitumor effect of SDT, histological staining was performed to analyze the tumor 3 days after treatment (Figure 6). H&E stain was used to define the gross tumor boundary. For the case of MRgFUS with 5-ALA, an obvious lack of Ki67 can be seen in the sonicated area, which is indicative of less proliferative tumor cells. In addition, there was strong TUNEL-positive expression at the same location which indicates apoptosis within the sonicated region. In contrast, MRgFUS alone did not show any damage in the sonicated tumor area. This indicates that the tumor-killing effect results from the interaction between ultrasound and the sonodynamic agent.

## Discussion

We have demonstrated that an SDT regimen of 5-ALA combined with MRgFUS at 5.5 W/cm^2^ (I_SPTA_) for 20 min can significantly inhibit tumor growth and prolong survival in an intracranial rat glioma tumor model in the absence of thermal dose. In addition, the survival was further improved when utilizing a multi-point MRgFUS sonication scheme compared to a single point sonication over the same total treatment duration. This extends and is in agreement with the earlier studies reporting the benefits of combing FUS with 5-ALA for brain tumor treatments^15^.

We did not only investigate the therapeutic effects of SDT with MRgFUS but also concurrently monitored the temperature change within the brain tumor using MRI thermometry with two different core body (rectal) temperatures. The maximum temperature elevation was 2.5 ± 1.0 °C and 3.3 ± 1.2 °C for 32 °C and 37 °C core body temperatures respectively during 20 min of MRgFUS at an I_SPTA_ of 5.5 W/cm^2^. The possible mechanisms of the drug activation in SDT are ultrasound induced temperature elevation, mechanical tissue motion or gas bubble collapse resulting in free radical formation However, the temperature elevation induced by FUS upon 32 °C and 37 °C core body temperatures could only cause less than 0.5 CEM43 thermal dose (see Table 1) but still achieve the therapeutic efficacy. Moreover, no significant difference can be found between these two body temperatures in the tumor growth and survival results. This indicates that thermal effects are unlikely explanation for the SDT. Therefore, the proposed mechanism from SDT at these low intensities is not thermal but mechanical interaction of ultrasound and the sonodynamic agent 5-ALA within the tumor possibly via collapsing gas bubbles^17^.

Histological analysis of the tumor tissue revealed reduced proliferation and increased apoptosis within the sonicated region. These results do not, however, rule out potential improvements in tumor control with the combination of SDT and hyperthermia at clinically relevant thermal doses (>10 min at 43 °C^18^).

SDT has been applied effectively to locally treat tumor tissue via both thermal and nonthermal effects^5,16,19,20^. However, once the temperature rises up to the level of hyperthermia (41 °C to 45 °C) bioeffects such as increased local blood flow, tumor vessel permeability, and cell metabolic rate, may result in synergistic effects and thus SDT and hyperthermia might lead to improved therapeutic efficacy^21^. It has been reported *in vitro* that hyperthermia caused by ultrasound may play an important role in the sonodynamic effect^16^, further *in vivo* works to this end, however, need to be conducted.

A major advantage of SDT is that it selectively targets the tumor volume via passive accumulation of the sonosensitizers through the inherently leaky angiogenic tumor vessels. For example, 5-ALA does not penetrate much the blood-brain barrier (BBB) by itself but can accumulate within a tumor^22^ which has been used clinically in the past for intraoperative tumor visualization. This property also allows the sonodynamic effect to occur specifically in the tumor which avoids damaging the normal brain tissue if the ultrasound parameters are selected appropriately. Certain brain tumors proliferate around healthy vessels with an intact BBB which will not be amenable to the passive accumulation of sonosensitizers. FUS in conjunction with ultrasound contrast agent (microbubbles) can transiently and reversibly open the BBB in not only preclinical studies^23^ but also clinical trials^24^. Hence, the feasibility of combining ultrasound-induced BBB opening and sonodynamic therapy needs further investigation.

It has been reported that SDT can produce BBB disruption and brain tissue damage in the absence of microbubbles if the applied intensity is high (~100 W/cm^2^)^19^. A similar study has examined the intensity of 10 W/cm^2^ in conjunction with 100 mg/kg 5-ALA at 1.04 MHz for 5 minutes to treat an intracranial rat glioma model^25^. In this study, we tested four different intensities from 2.8 to 22 W/cm^2^ (Figure 1) in the normal rat brain and found out that the highest intensity of 22 W/cm^2^ for 20 min caused a thermal lesion after sonication; therefore, we chose 5.5 W/cm^2^ as our experimental intensity in this study as it is close to the reported effective literature values^15,25^ and the thermal temperature alteration does not reach the hyperthermia regime.

SDT containing the target tissue with the sensitizer accumulated and ultrasound exposure^26^, allows the treatment to overcome the shortcomings of PDT in cancer. 5-ALA, a potent sonosensitizer, is intracellularly converted into protoporphyrin IX (PpIX) and accumulates in the heme biosynthesis pathway of cell mitochondria^27^. Previous reports have demonstrated ALA-SDT produces antitumor effects on pancreatic cancer^28^, melanoma^29^, osteosarcoma^30^, tongue squamous carcinoma^31^, and glioma^15,25^. It has been shown that SDT could generate intracellular reactive oxygen species (ROS) and subsequently produce direct cytotoxicity in malignant cells^26^. Oxidative stress has been linked to increased intracellular ROS levels that cause damage to deoxyribonucleic acid (DNA), proteins and lipids^32^. Cheng *et al*.^33^ demonstrated that 5-aminolevulinic acid (5-ALA)- induced PpIX which are mainly located on the mitochondria, can induce THP-1 macrophage apoptosis by generating a large amount of ROS in mitochondria after the production of sonoluminescence. Based on the previous study^15^, the maximum accumulation of PpIX in the brain tumor was 2–8 h after 5-ALA administration; hence, the time-point for executing SDT after 5-ALA administration was reasonably designed to be 6 h.

In the present results, multiple-point treatment has a better therapeutic outcome than single-point even with the same total exposure duration. More sonosensitizers can be activated by ultrasound exposure with a larger sonicating area, which correlates with an improved treatment. In addition, the optimized exposure duration still needs to be determined on the given dosage of drugs.

In the clinical context, transcranial MRgFUS of brain tissue is limited by the presence of the skull. Once thought an impossible barrier, sophisticated phased array ultrasound transducers have been shown to be capable of reconstructing the acoustic focus through the human skull^34^. Clinical protocols aimed at elevating diseased brain tissue to ablative levels (>55 °C) are constrained to the center of the brain with short and high power sonications due to the absorption of the skull bone in the path of the ultrasound beam^35^. Utilizing pulsed-wave ultrasound and the phased-array system to avoid skull over-heating allows this technology to be advanced towards clinical applications^13,36^. The order of magnitude reduction in the applied intensity when compared to thermal ablations may make these treatments not only feasible but also in off-center targets where ablation is not feasible today. However, the frequency used in this study (1.06 MHz) is significantly higher than the optimal frequency used in the clinical ablation treatments (approximately 650 kHz^37^) and would be a limitation in a clinical setting even with the reduced power requirement. Hence, if the results could be extended to the current trans-skull treatment frequency range, then the method may make the tumor treatments throughout the skull cavity feasible in humans.

To conclude the current results, the combination of 5-ALA and FUS sonication did affect the tumor growth by inducing the cytotoxic effects. However, the thermal effect of the initial body temperature does not make difference in the outcome of the treatment. The survival data is more promising with multiple points treatment. The next step of this experiment is to further explore the acoustic parameters and optimize the treatment efficacy.

## Materials and Methods

All animal procedures were carried out with the prior approval of the Sunnybrook institutional animal care committee, and in accordance with the guidelines of the Canadian Council on Animal Care.

### Preparation of 5-aminolevulinic acid

The sonosensitizing agent 5-aminolevulinic acid (5-ALA, Levulan^®^, DUSA Pharmaceuticals, Inc., Wilmington, MA 01887, USA) was tightly sealed in a brown bottle, protected from light and stored at 25 °C. Before use, the agent was dissolved in phosphate-buffered saline (PBS, pH 7.4) at a concentration of 60 mg/mL and then stored at 4 °C in the dark. A dose of 60 mg/kg 5-ALA was administrated intravenously 6 hours before sonication.

### *In vivo* C6 glioma brain tumor model

C6 glioma brain tumor cells (ATCC^®^ CCL-107^TM^) were cultured in F-12K medium (Kaighn’s Modification of Ham’s F-12 Medium) supplemented with 2.5% heat-inactivated fetal bovine serum (FBS) and 15% horse serum in 10 cm tissue culture plates in a 5% CO_2_-containing incubator at 37 °C. Cell number and viability were calculated with a hemocytometer via trypan blue exclusion.

A total of 37 male Sprague-Dawley rats (Taconic Biosciences, Germantown, NY, USA), that ranged in weight from 280 to 400 g, were used in this study. The animals were housed in the Sunnybrook Research Institute animal facility (Toronto, ON, Canada) and allowed free access to food and water. The rats were anesthetized by 2.5% isoflurane inhalation during the tumor implantation surgery. A total of 4 × 10^5^ C6 tumor cells suspended in 10 μL of phosphate buffered saline (PBS) and Matrigel (Corning^®^ Matrigel^®^, Discovery Labware, Inc., MA 01730) were slowly injected into the right cortex (0.5 mm anterior and 2.5 mm lateral to the bregma at a depth of 1.5 mm from the dura). After injection, the needle stayed in the brain for 5 min and was slowly withdrawn over another 1 min. The skin incision was then sewn up with 5–0 polydioxanone sutures.

### Experimental grouping

The experiments in this study consisted of two parts: 1) safety evaluation of sonication parameters during SDT in normal rat brain and 2) therapeutic efficacy of SDT in an intracranial rat brain tumor model. First, four different *in situ* spatial-peak temporal-average acoustic intensities (I_SPTA_), namely 2.8 W/cm^2^, 5.5 W/cm^2^, 11 W/cm^2^, and 22 W/cm^2^ were applied trans-cranially in four normal rat brains in order to determine the acoustic intensity threshold for activating the sonosensitizer. Intensity estimates were based on an assumed through-skull transmission of approximately 64% at 1.06 MHz^38^ and propagation through 5 mm of brain tissue with an attenuation coefficient of 5 Np/m/MHz^23^. A dose of 60 mg/kg 5-ALA was intravenously injected through the rat tail vein 6 hours before sonication. Real-time MR thermometry was acquired to monitor the temperature response during sonication and T2-weighted MR images were used to detect brain tissue damage after treatment on day 3 and day 7 post-treatment.

To examine the therapeutic effect of SDT at different resting core body temperatures and to explore the strategy of using multi-point sonications for SDT, brain tumor-bearing rats were randomly divided into six groups as follows: 1) control (n = 5), 2) 5-ALA alone (n = 5), 3) FUS alone (n = 5), 4) 5-ALA + FUS at 32 °C core body temperature (5-ALA + FUS@32 °C, n = 5), 5) 5-ALA + FUS at 37 °C core body temperature (5-ALA + FUS@37 °C, n = 5), and 6) 5-ALA + FUS with multi-point sonications (5-ALA + FUS@37 °C_MPs, n = 6). A dose of 60 mg/kg 5-ALA was injected as a bolus approximately 6 hours before sonication. The treatment was performed 7 days following tumor implantation when the tumor had reached a maximum diameter of 1–3 mm as determined by contrast-enhanced T1-weighted MR images. The endpoint of the experiment was set to be 20% of body weight loss or abnormal animal behavior.

### MRI-guided FUS treatment

Prior to sonication rats were anesthetized with 2% isoflurane inhalation with medical air as the gas carrier. The hair on the top of their head was removed with clippers and depilatory lotion. The rats were then placed supine on a MR-compatible sled and their head was coupled to a tank of degassed water with ultrasound gel. The core temperature of the rats was set by using a temperature controlled water blanket (T/Pump, Stryker, MI, USA) and measuring the rectal temperature. The sonications were not started until the target core temperature was reached.

**Figure 7:**
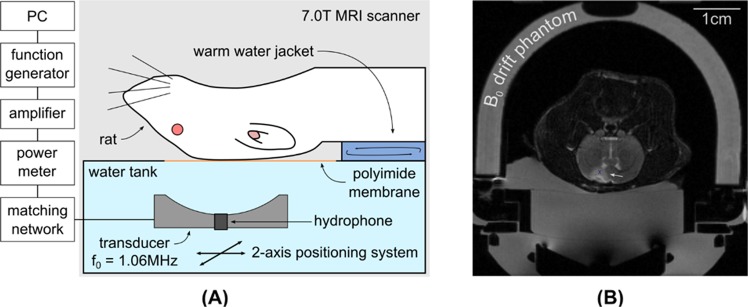
(**A**) Experimental setup of MRI-guided focused ultrasound sonodynamic therapy in a rat brain tumor model, each rat was placed in a supine position and the ultrasound is focused through the intact skull on a target region within the tumor in the rat brain. (**B**) A T2-weighted image in the axial plane of the rat brain, the heavy mineral oil phantom used for B_0_ drift correction is visible towards the edge of the field-of-view. The position of the acoustic focus is marked with a blue ‘x’ and the hyper-intense tumor location is indicated by the white arrow.

Continuous wave sonication in the rat brain was achieved using a single-element spherically-curved transducer (resonant frequency, f_0_ = 1.06 MHz, focal number = 0.8, diameter = 25 mm, central fenestration = 4 mm) with an acoustic efficiency of 65% measured using a radiation force balance technique with a laboratory balance (AE200; Mettler Instruments, Hightown, NJ) as the force detector^39^. The spherically curved transducer transmits a converging wave that forms a focus close to the center of the radius of curvature, and it was mounted within the MRI-compatible preclinical focused ultrasound system (In-house developed prototype of RK-300; FUS Instruments Inc., Toronto, ON, Canada) with a 2-axis motorized stage to allow precise anatomical targeting of the focal region. The MRI-compatible sled was coupled to the water tank housing the transducer during sonication. The spatial coordinates of the FUS positioning system were co-registered to a 7.0 T small-bore preclinical MRI scanner (BioSpin 70/30, Bruker, Billerica, MA, USA). The experimental setup is shown in Figure 7A. T1-weighted images were obtained immediately following intravenous injection of a gadolinium-based contrast agent (0.2 mmol/kg, Gadovist, Schering AG, Berlin, Germany) to confirm the tumor location. This enabled the targets of sonication to be chosen in the software based on the MR images of the anatomy.

The selected acoustic power for SDT was 0.32 W and the estimated I_SPTA_ was 5.5 W/cm^2^ which corresponds to an estimated *in situ* peak pressure amplitude of 420 kPa in the focus. The total ultrasound exposure duration was 20 min for both the single point group and the multi-point sonication group. A fast low angle shot (FLASH) gradient-echo sequence was used for thermometry (temporal resolution = 5 s) and both the real and imaginary parts of the MR image data were used to process the temperature maps as in^40,41^ using the proton resonance frequency shift method. The MR imaging parameters are shown in Table 2. Magnetic field drift was corrected by subtracting the phase change measured from a heavy mineral oil phantom that was placed within the imaging field-of-view^42^ as shown in Figure 7B. The accumulated thermal dose in the tumor was calculated in cumulative equivalent minutes at 43 °C (CEM43) using the Sapareto-Dewey time-temperature equation^43^.

**Table 2:** MR Imaging Parameters.

Parameter	T1-weighted sequence	T2-weighted sequence	Thermometry sequence
Sequence type	RARE	RARE	FLASH
Echo time (ms)	10	70	10
Repetition time (ms)	500	4000	50
Echo train length/RARE factor	2	10	1
Field-of-view (cm)	6	6	6
Matrix	150 × 150	200 × 200	100 × 100
Slice thickness (mm)	1.5	1.5	3

RARE = Rapid acquisition with relaxation enhancement, FLASH = Fast low angle shot.

### Histology and immunochemistry staining

To confirm the cytotoxic effect of SDT, tumor-bearing rats were perfused with saline and then 4% paraformaldehyde 72 hours after the treatment. Brains were harvested and fixed in 4% paraformaldehyde for 48–72 hours prior to transfer to 70% alcohol. Afterwards, the brains were consecutively sliced to a thickness of 5 µm. Hematoxylin-eosin (H&E) stain was used for gross histological examination. For immunohistochemical analysis, the brain slices were treated with 3% hydrogen peroxide to block endogenous peroxide activity before incubation with the primary antibody. After blocking for 1 hour in 4% non-fat milk containing 1% Triton X-100, the slices were incubated with the Ki67 primary antibody (1:700; ab15580, Abcam, Cambridge, MA, USA). After a brief wash, slices were processed with a rabbit specific HRP/DAB system (ab80437, Abcam) to visualize Ki67 expression, which indicated tumor proliferation.

Tumor sections were simultaneously processed with the TUNEL assay (DeadEnd™ Colorimetric TUNEL System, Promega, Madison, WI, USA) following the manufacturer’s instructions to identify the apoptotic area in the tumor. Briefly, the slides were fixed with 4% formaldehyde and permeabilized with 0.2% Triton X-100 in PBS. The slides were labeled with a TdT reaction mixture for 60 minutes at 37 °C and then blocked by immersing slides in 0.3% hydrogen peroxide. Next, 100 µL of streptavidin (HRP) was added to bind the tissues for 30 min and finally 100 µL of DAB was used to stain the tissues until the brown color became visible.

### Statistical analysis

All values are displayed as mean ± standard deviation (SD). The results were analyzed with one-way analysis of variance with the post hoc Dunnet test and the survival data were analyzed by log-rank test. All *p*-values were two-sided and statistical significance was defined as *p* < 0.05. Calculations were processed on a computer using SPSS version 20.0 (SPSS Inc., Chicago, Illinois, US).

## Data Availability

Original data are available from the authors upon request.
